# Formation of Nb(C,N) Carbonitride in Cast Austenitic Heat-Resistant Steel during Directional Solidification under Different Withdraw Rates

**DOI:** 10.3390/ma11122397

**Published:** 2018-11-28

**Authors:** Yinhui Zhang, Jian Yang

**Affiliations:** 1 State Key Laboratory of Advanced Special Steel, School of Materials Science and Engineering, Shanghai University, Shanghai 200444, China; 2 State Key Laboratory for Advanced Metals and Materials, University of Science and Technology Beijing, Beijing 100083, China

**Keywords:** austenitic steel, carbonitride, directional solidification, growth rate, morphology

## Abstract

It is recognized recently that primary “Chinese-script” Nb(C,N) carbonitride is critical to the development of cast austenitic heat-resistant steels for ultra-high temperature applications. In this paper, the precipitation behavior of Nb(C,N) carbonitride in a novel creep and fatigue resistant steel was investigated by the use of the liquid metal cooling directional solidification (LMC-DS) method under different withdraw rates. Thermodynamic calculations were also performed to aid in the understanding of the solidification behavior. Microstructural characterization and thermodynamic calculation agreed that the alloy solidified in the path of primary austenite, eutectic Nb(C,N) carbonitride, and secondary ferrite, regardless of the withdraw rate. However, the primary and secondary dendrite arm spacing decreased significantly with an increase in the withdraw rate, and a quantitative relationship was established. Furthermore, the eutectic reaction range increased at a higher withdraw rate, due to the rapid increase of the solid phase fraction and the accumulation of solutes in the interdendritic liquid phase. This gave rise to a decline in the interlamellar spacing of primary Nb(C,N) carbonitride sheets and rods for the higher withdraw rate. Therefore, a fine “Chinese-script” Nb(C,N) carbonitride in this type of alloys can be achieved through increasing the withdraw rate or the cooling rate during casting.

## 1. Introduction

In recent years, more strict environmental and fuel consumption regulations have been placed worldwide, aiming at reducing exhaust gas emissions and preventing global warming [1]. For the automotive industry, gas emissions can be reduced by increasing the operating temperature and pressure of automotive engines, with a further benefit of increasing the engine power [2]. The gas temperature of gasoline engines now reaches as high as 1050 °C, approximately 200 °C higher than the conventional gas temperatures [3]. This results in many failure events of exhaust manifolds and turbine housings in automotive exhaust systems, made by incumbent materials (SiMo cast iron and Ni-resist D5S, etc.) [4,5]. Therefore, automotive industries are in need of developing novel and cost-effective alloys for such ultra-high temperature applications.

Candidate structural materials of interest comprise ferritic and austenitic heat-resistant steels with superior mechanical properties and oxidation resistance at high temperatures, as well as Ni-based superalloys [1,6]. Because of the complex shape of the exhaust components, the cast grade of these materials are specially demanded [5]. A new family of Nb-bearing cast austenitic heat-resistant steels is currently under development by the authors [7,8,9,10,11]. These alloys exhibit much better creep properties than that of commercial alloy 1.4826 at 1000 °C, owing to the precipitation of primary and secondary Nb(C,N) carbonitride [12]. Particularly, the primary “Chinese-script” Nb(C,N) carbonitride can prevent grain boundary sliding and strengthen the interdendritic regions, thus improving the creep property more effectively than the blocky carbonitride [13]. Furthermore, this “Chinese-script” Nb(C,N) carbonitride, rather than the blocky one, can improve the thermo-mechanical fatigue properties, since it delays the nucleation and propagation of fatigue cracks [11,14]. Therefore, a key demand for creep- and fatigue-resistant cast austenitic heat-resistant steels is to form fine “Chinese-script” Nb(C,N) carbonitride during casting.

The formation of primary Nb(C,N) carbonitride associates profoundly with the composition and casting of these austenitic alloys. Extensive studies on the compositional effects in Ni-based superalloys and austenitic steels have established that C, Nb and Y/Ce additions improve the formation of “Chinese-script” MC-type carbide, whilst Ti and Hf additions accelerate faceted and blocky MC-type carbide precipitation [15,16,17,18,19,20,21,22]. Our previous studies have recognized that N additions will transform this carbonitride morphology from “Chinese-script” to blocky features as well [23]. However, N is a powerful austenite stabilizer, which can substitute Ni, a common but relatively expensive austenite stabilizer, thereby reducing the cost [24]. It is therefore required that the morphology of Nb(C,N) carbonitride is controlled by optimizing the casting process.

Several studies on Ni-based alloys have highlighted that MC-type carbide is irregularly shaped for high cooling rates, and spheroidized for low cooling rates [16,25,26]. For austenitic steels, Buchanan et al. has recently recognized that the morphological difference of MC-type carbide is determined by the nucleation sites: on the existing austenite dendrite, or in the liquid phase ahead of the liquid–solid (L/S) interface, or on Ti-rich spheres in the liquid phase [27,28]. However, the formation mechanism of Nb(C,N) carbonitride has not been investigated thoroughly, particularly in cast austenitic heat-resistant steels with relatively high Nb contents.

Therefore, the aim of the current research is to investigate the precipitation behavior of Nb(C,N) carbonitride under different solidification conditions. For this purpose, a novel cast austenitic heat-resistant steel with a good combination of creep, fatigue, and oxidation resistance was deliberately selected from our alloy system. The liquid metal cooling directional solidification (LMC-DS) method was applied to offer real microstructural developments during solidification. In the presence of the LMC-DS system, the temperature gradient was kept constant, whilst the withdraw rate varied from 20 to 50 µm/s. Computational thermodynamic calculations were also performed to aid in the interpretation of the solidification behaviors. Based on these experimental and simulation methods, the solidification path of this alloy was identified, and a relationship between the microstructure and withdraw rate was established. The generated data and understanding will help to optimize the casting process of this type of alloy, and to improve their ultra-high temperature mechanical properties.

## 2. Materials and Methods

A novel cast austenitic heat-resistant steel named alloy 3C2N was studied in the current research. This alloy was melted in a vacuum induction furnace, and cast into a cylindrical ingot with a diameter of 80 mm, weighing about 20 kg. The chemical composition was quantitatively analyzed by inductively coupled plasma atomic emission spectrometry (ICP-AES) for Ni, Mn, Nb, and P, ammonium persulfate oxidation titration for Cr, infrared absorption for C and S, conductivity for N, and gravimetric method for Si, and it is summarized in Table 1. The ingot was machined into directional solidification (DS) bars with lengths and diameters of 100 and 6 mm, respectively. These DS bars were then mechanically ground by SiC emery papers up to 600 grit prior to the DS experiments.

**Figure 1:**
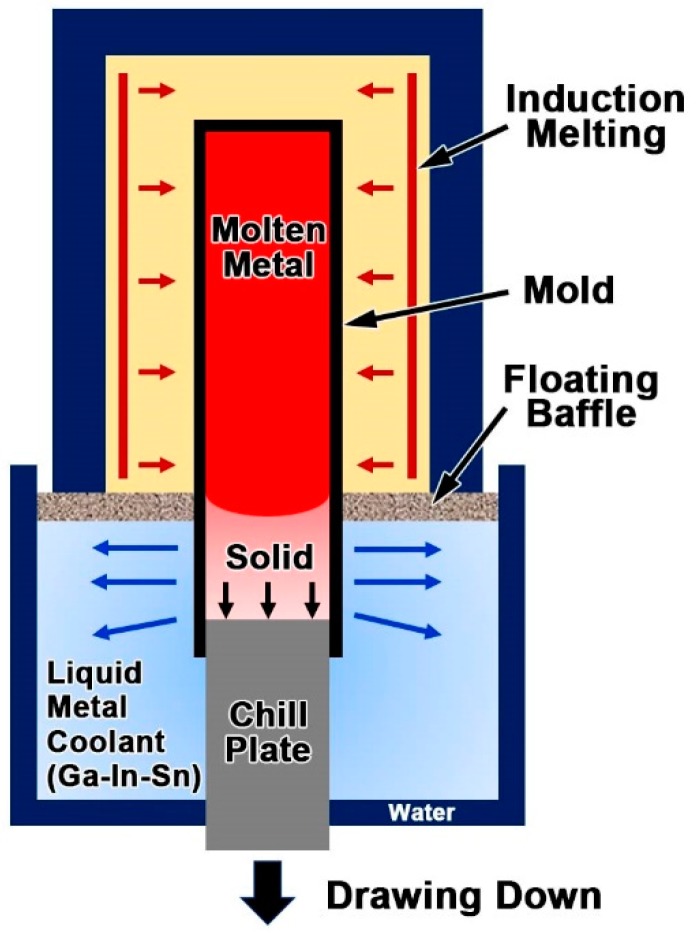
Schematic illustration of the LMC-DS processing [10].

LMC-DS technology, an effective method for solidification investigation [10,23], was applied in the current research. This technology is based on the Bridgman-type DS concept that the DS bar is withdrawn from the hot zone to the cold zone at a controlled speed, so that the L/S interface resides within the transition region (mushy zone) [29]. Different from the traditional Bridgman type DS technology that relies on relatively inefficient radiation cooling, the LMC-DS technology utilizes a low-melting-point, liquid–metal coolant in the cold zone to extract heat more efficiently, and therefore, the solidification microstructure can be well preserved. Figure 1 shows the schematic illustration of the LMC-DS processing [10]. The DS bar was melted in the graphite induction furnace, with a maximum heating temperature of about 2000 °C, and a vacuum degree of 10^−4^ Pa. It then moved vertically under withdraw rates of 20 and 50 µm/s, and solidified under a temperature gradient of 10 °C/mm, controlled by a W-Re5/26 thermocouple [30]. After directional solidification to a constant length of 50 mm, the DS bar was quenched immediately by a rapid displacement of the alumina crucible into the liquid metal bath (Ga-In-Sn).

Subsequently, the DS bars were sectioned longitudinally to investigate the quenched interfacial morphology and microstructural development during the LMC-DS process. The specimens for macro-grain structure observation were mechanically ground by SiC emery papers up to 1000 grit, prior to etching in a solute of 20% H_2_O_2_ + 40% HCl + 40% H_2_O. The specimens for microstructure analyses were mechanically ground as well, but up to 3000 grit, and then polished by 0.1 μm diamond powder without etching. The macro-grain structure was examined by the use of an office scanner, whilst the microstructure was characterized utilizing an Axio Imager A2m optical microscope (OM, ZEISS Ltd., Oberkochen, Germany) and a SUPRA 55 field emission-scanning electron microscope (FE-SEM, ZEISS Ltd., Oberkochen, Germany), operated in backscattered electron (BSE) imaging mode. The composition of the liquid phase in the mushy zone was determined by a JXA-8530F field emission electron probe microanalyzer (FE-EPMA, JEOL Ltd., Tokyo, Japan), equipped with five wavelength-dispersive spectrometers (WDS).

Thermodynamic calculation was also carried out to aid in the interpretation of the solidification behavior of alloy 3C2N, by the use of the JMatPro software (version 5.0, Sente Software Ltd., Guildford, UK) with a stainless-steel database. The phase diagram was calculated under the equilibrium condition, whilst the solidification sequence of different phases was calculated under the Scheil–Gulliver condition. The measured chemical composition listed in Table 1 was used for these calculations.

## 3. Results

### 3.1. Thermodynamic Calculations

**Figure 2:**
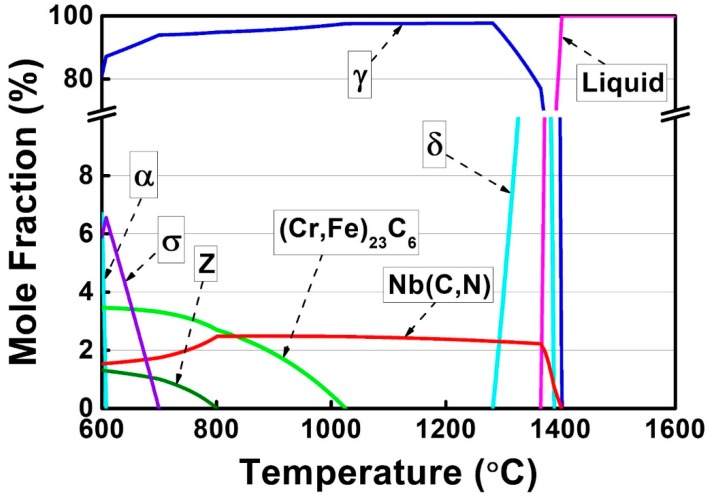
Phase diagram of alloy 3C2N calculated by JMatPro software under the equilibrium condition.

**Figure 3:**
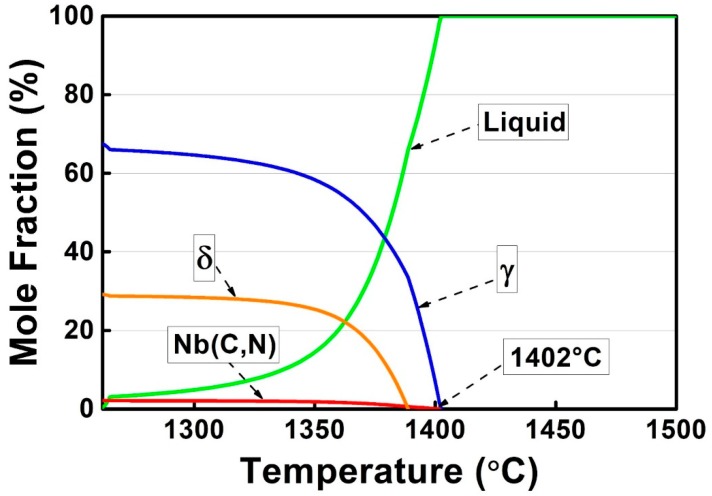
Mole fractions of different phases that formed during the liquid–solid solidification of alloy 3C2N, calculated by JMatPro software under the Scheil–Gulliver condition.

In order to predict the microstructure of as-cast alloy 3C2N, its phase diagram was calculated by using JMatPro software under the equilibrium condition, and plotted in Figure 2. It predicted that the microstructure of as-cast alloy 3C2N consisted of Nb(C,N) carbonitride and a small amount of (Cr,Fe)_23_C_6_ carbide on the austenitic matrix at a service temperature of 1000 °C. This Nb(C,N) carbonitride would transform into the Z-phase (CrNbN) as the temperature decreased below 800 °C, along with the formation of (Cr,Fe)_23_C_6_ carbide, σ-phase, and ferrite. However, these equilibrium phases at lower temperatures might not form in the as-cast microstructure, due to rapid casting processing. Therefore, the phases that formed during the liquid–solid reaction and resided in as-cast microstructure were simulated by the JMatPro software under the Scheil–Gulliver condition, and plotted in Figure 3. The phases were predicted to solidify in the sequence as:

L → L + γ → L + γ + Nb(C,N) → L + γ + Nb(C,N) + δ → γ + Nb(C,N) + δ(1)

A relatively high fraction of δ-ferrite was retained at the end of solidification, different from that predicted in the equilibrium phase diagram. Besides, no (Cr,Fe)_23_C_6_ carbide was predicted in this liquid-solid reaction stage. It is thus presumed that the microstructure of the as-cast alloy 3C2N was composed of Nb(C,N) carbonitride and residual δ-ferrite on the austenitic matrix.

### 3.2. Microstructure of the as-Cast Ingot

**Figure 4:**
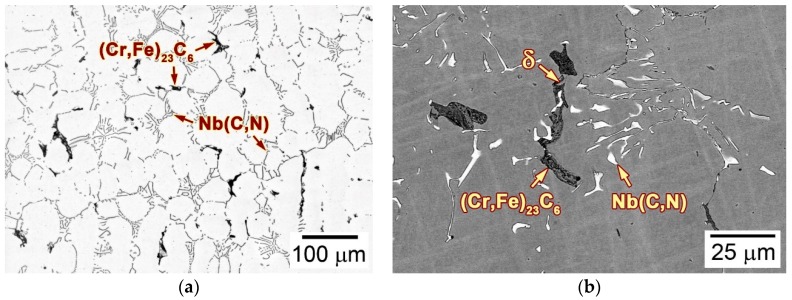
(**a**) Optical and (**b**) scanning electron microscope-backscattered electron (SEM-BSE) images showing the microstructure of as-cast alloy 3C2N.

The microstructure of the as-cast alloy 3C2N was characterized before LMC-DS processing. Figure 4a,b are optical and scanning electron microscope-backscattered electron (SEM-BSE) images showing the typical microstructure of the as-cast ingot at different magnifications. Figure 4a shows that the microstructure was composed of austenitic dendrites and duplex interdendrites, along with some black-contrast vermicular structures decorating the grain boundaries. Figure 4b shows that these duplex interdendrites consisted of lamellar precipitates with white contrast and interlamellar austenites, exhibiting the “Chinese-script” morphology. The vermicular structure comprised two type of precipitates: the cellular precipitate with black contrast, and the blocky precipitate with gray contrast (Figure 4b). Scanning electron microscope-energy dispersive spectrometer (SEM-EDS) analyses indicated that the lamellar precipitate was enriched in Nb, C, and N, whilst the vermicular structure was enriched in Cr and Fe. These precipitates were further identified by transmission electron microscope (TEM) and corresponding selected area diffraction pattern (SADP) analyses, confirming the white-contrast precipitate as Nb(C,N) carbonitride, the black-contrast one as (Cr,Fe)_23_C_6_ carbide, and the gray-contrast one as residual δ-ferrite.

### 3.3. Microstructural Developments during Directional Solidification

#### 3.3.1. Macro-Grain Structures

**Figure 5:**
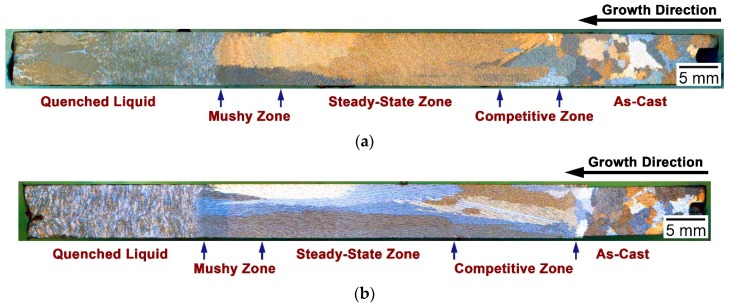
Optical images of longitudinal macro-grain structures in as-cast alloy 3C2N after the LMC-DS processing under different withdraw rates: (**a**) 20 and (**b**) 50 µm/s.

The LMC-DS processing was adopted in the current research to investigate the solidification behavior of as-cast alloy 3C2N under different withdraw rates. The macro-grain structures after the LMC-DS processing are shown in Figure 5a,b from the longitudinal view. The DS bar exhibited several distinct grain zones at the withdraw rate of 20 µm/s (Figure 5a), corresponding to different solidification stages during the LMC-DS process. These grain zones were denoted as the as-cast zone, the competitive zone, the steady-state zone, the mushy zone, and the quenched liquid zone, in order of solidification sequence. The as-cast zone referred to the region that was not melted during the LMC-DS process, and thus, the coarse equiaxed grains were retained. The competitive zone consisted of fine columnar grains with different growth directions, whilst the steady-state zone revealed much coarser columnar grains with the growth direction being parallel to the temperature gradient direction. The mushy zone indicated the region where liquid and solid phases co-existed during the LMC-DS process, and the detailed microstructural characterization is presented in Section 3.3.2. The average columnar grain length and width within the mushy zone and the steady-state zone were measured to be 22.3 ± 6.1 and 2.4 ± 0.7 mm at a withdraw rate of 20 µm/s. After directional solidification to the constant length of 50 mm, the DS bar was quenched in the liquid metal bath, thus forming extremely fine equiaxed grains in the quenched liquid zone.

At a withdraw rate of 50 µm/s, the DS bar solidified in order of the similar five distinct stages (Figure 5b), but showing different solidification widths and columnar grain sizes from that at 20 µm/s. When increasing the withdraw rate, the competitive zone expanded, while the steady-state zone contracted during the LMC-DS process, except for the mushy zone, which remained constant. Moreover, the number of columnar grains increased within the mushy zone and the steady-state zone, with the average columnar grain length and width measured to be 21.5 ± 8.2 and 1.7 ± 0.6 mm, respectively. Analyzing such change requires detailed microstructural characterization of each solidification zone in these DS bars under the two withdraw rates.

#### 3.3.2. Microstructures

**Figure 6:**
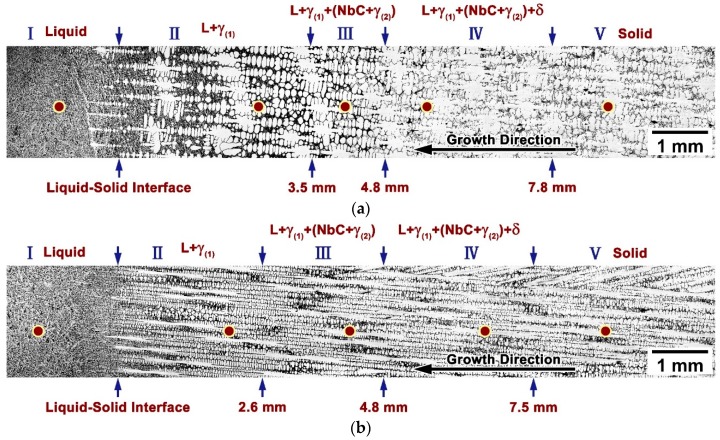
Optical images of longitudinal microstructures of the mushy zones in as-cast alloy 3C2N after the LMC-DS processing under different withdraw rates: (**a**) 20 and (**b**) 50 µm/s.

The mushy zones of the DS bars were characterized to elucidate the effect of the withdraw rate on the solidification behavior of as-cast alloy 3C2N during the LMC-DS process. Figure 6 shows the longitudinal microstructures of the mushy zones in as-cast alloy 3C2N under the withdraw rates of 20 and 50 µm/s. The solidification locations with solid-phase fractions of 0 and 100%, known as the L/S interface and the quenching interface, corresponded to the initiation and termination of the mushy zones in the current research. Their temperatures (i.e., the liquidus and solidus temperatures) were measured to be 1428 and 1356 °C by DSC analyses, respectively. The temperature gradients of the two DS bars were then calculated to be 9.2 and 9.6 °C/mm by measuring the temperature range and width of the mushy zones, approximating to the constant (10 °C/mm) of the DS system.

The mushy zone withdrawn at 20 µm/s exhibited that the primary austenitic dendrites aligned themselves well in the direction of the temperature gradient, and grew towards the molten metal (Figure 6a). The primary dendrite arm spacing (PDAS) was determined to be 298.2 ± 44.3 µm at the lower positions of the mushy zone. The secondary dendrite arms were orthogonal to the primary arms, with the spacing (SDAS) being determined to be 88.8 ± 9.5 µm. The residual liquid phase in the interdendritic regions was gradually consumed to form γ-austenite, Nb(C,N) carbonitride, and δ-ferrite. Particularly, the Nb(C,N) carbonitride was observed to nucleate and grow through the eutectic reaction:

L → Nb(C,N) + γ_(2)_ (2)

Therefore, the mushy zone can be divided into five different liquid–solid reaction stages, comprising the full liquid stage (stage I), the primary γ-austenite solidification stage (stage II), the eutectic reaction stage (stage III), the interdendritic δ-ferrite solidification stage (stage IV), and the complete solid stage (stage V). The initiation location of each reaction stage was marked in Figure 6a as well.

With an increase in the withdraw rate to 50 µm/s, the primary austenitic dendrites still paralleled the direction of the temperature gradient in the mushy zone, and grew in the order of the same five reaction stages (Figure 6b). The PDAS was determined to be 152.4 ± 14.0 µm, approximately half of that at 20 µm/s. The secondary dendrites were significantly finer as well, with the SDAS determined to be 42.3 ± 3.1 µm. Moreover, the Nb(C,N) carbonitride precipitated at higher positions of the mushy zone, thus expanding the eutectic reaction stage by 0.9 mm (about 9 °C).

**Figure 7:**
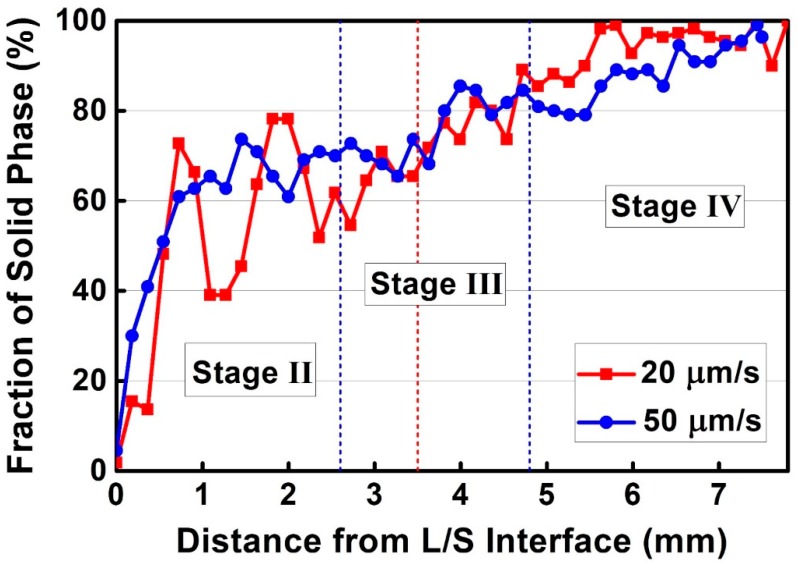
Volume fractions of solid phase in the mushy zones of as-cast alloy 3C2N during the LMC-DS process under withdraw rates of 20 and 50 µm/s.

Volume fractions of solid phase in the two mushy zones are plotted in Figure 7 as a function of distance from the L/S interface. When the temperature decreased below the liquidus temperature, solidification started with a sharp increase in the volume fraction of solid phase. Two obvious peaks were observed on the volume fraction cure withdrawn at 20 µm/s, corresponding well to the coarse, nodular dendrites in stage II, as shown in Figure 6a. The volume fraction increased continuously and slowly in stage III and IV. Comparing the two curves, it was agreed that the eutectic reaction occurred only when the volume fraction exceeded 65%.

**Figure 8:**
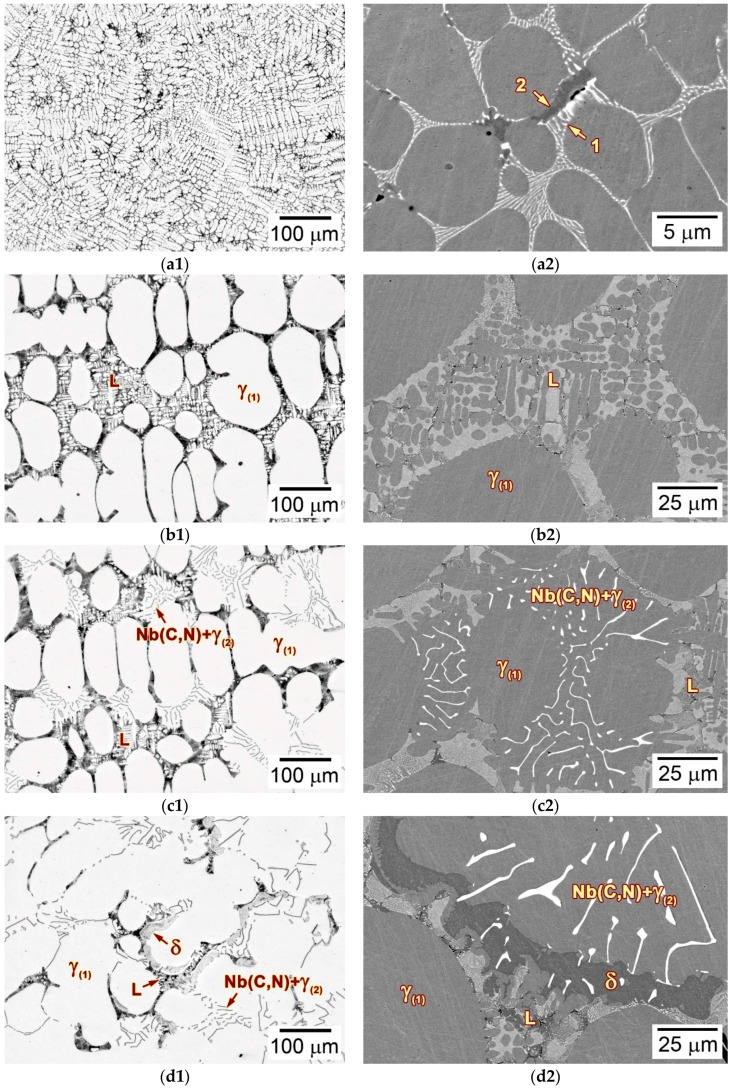

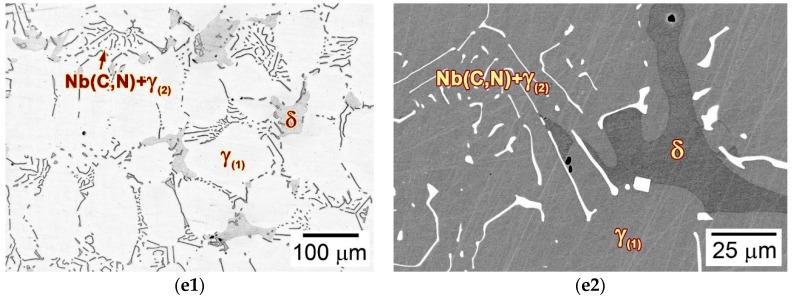
Optical and enlarged SEM-BSE images showing the typical microstructure of each solidification stage marked by red circles in Figure 6a under the withdraw rate of 20 µm/s: (**a1**,**a2**) stage I; (**b1**,**b2**) stage II; (**c1**,**c2**) stage III; (**d1**,**d2**) stage IV; (**e1**,**e2**) stage V.

Typical locations at all solidification stages in Figure 6a,b were analyzed in detail to examine the solidification path of as-cast alloy 3C2N. Figure 8 shows the typical microstructure of each solidification stage marked by red circles in Figure 6a at the withdraw rate of 20 µm/s. Stage I exhibited the extremely fine dendritic structure (Figure 8a1), in which the interdendritic constituent consisted of the white-contrast Nb(C,N) carbonitride (marked by “1”) and the black-contrast δ-ferrite (marked by “2”) (Figure 8a2). These fine precipitates, as well as the small dendritic structure were presumed to form during the final quenching process, whose withdraw rate was overwhelmingly higher than the steady withdraw rate (20 and 50 µm/s). At stage II, the γ-austenite solidified first and formed a relatively coarse dendritic structure (Figure 8b1). The liquid phase resided in the interdendritic regions, where it extended in all directions, thereby preventing the bridge-connection of dendrites. This residual liquid phase had a similar fine structure (Figure 8b2) to that in stage I, indicating that it was well preserved during the final quenching process. As temperature decreased to stage III, Nb(C,N) carbonitride started to nucleate and grow at the expense of the residual liquid phase (Figure 8c1). This was well-recognized as the eutectic reaction (Equation (2)), by which the Nb(C,N) carbonitride formed the typical “Chinese-script” morphology (Figure 8c2). After the eutectic reaction, δ-ferrite solidified by consuming the rest of the liquid phase, and exhibited a vermicular morphology in the interdendritic regions (Figure 8d1,d2). Stage V showed the complete solid structure consisting of the “Chinese-script” Nb(C,N) and the vermicular δ-ferrite (Figure 8e1). By comparison with the as-cast microstructure before the LMC-DS processing (Figure 4b), the size and quantity of the residual δ-ferrite increased obviously, without any (Cr,Fe)_23_C_6_ carbide precipitation (Figure 8e2).

**Figure 9:**
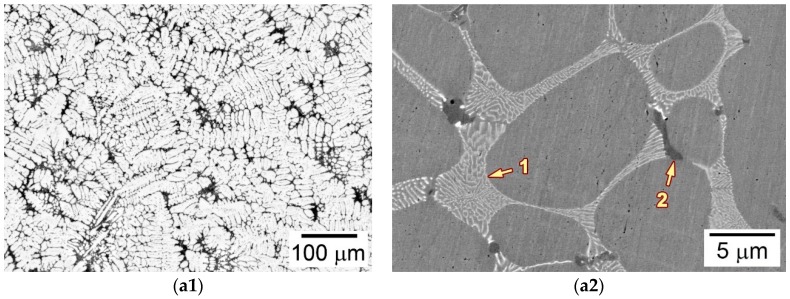

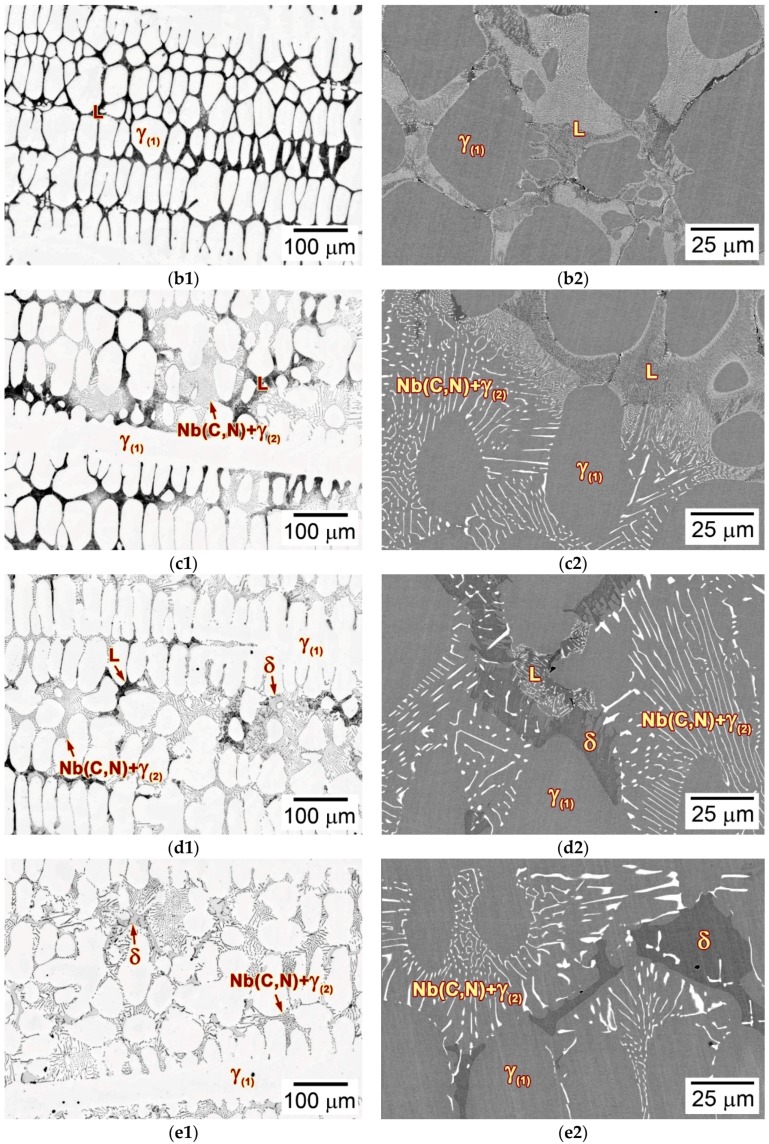
Optical and enlarged SEM-BSE images showing the typical microstructure of each solidification stage marked by red circles in Figure 6b under the withdraw rate of 50 µm/s: (**a1**,**a2**) stage I; (**b1**,**b2**) stage II; (**c1**,**c2**) stage III; (**d1**,**d2**) stage IV; (**e1**,**e2**) stage V.

Figure 9 shows the typical microstructure of each solidification stage marked by red circles in Figure 6b at the withdraw rate of 50 µm/s. Stage I revealed the fine dendritic microstructure (Figure 9a1), with the similar quenched SDAS as being 20 µm/s, as well as the fine Nb(C,N) carbonitride (marked by “1”) and δ-ferrite (marked by “2”) in the interdendritic regions (Figure 9a2). As solidification began in stage II, γ-austenite solidified primarily (Figure 9b1), and formed a much finer SDAS than that at 20 µm/s. The liquid phase flowed smoothly along the interdendritic regions, though the fraction of solid phase increased rapidly in this stage (Figure 9b1,b2). The eutectic reaction (Equation (2)) regarding the formation of Nb(C,N) carbonitride occurred subsequently in stage III by consuming the residual liquid phase (Figure 9c1). Comparing the lamellar Nb(C,N) carbonitrides forming at 20 and 50 µm/s, the interlamellar spacing was decreased significantly with an increase in the withdraw rate (Figure 8c2 and Figure 9c2). The solidification ended in stage IV, where δ-ferrite solidified at the expense of the last liquid phase in the interdendritic regions, and formed a vermicular morphology (Figure 9d1,d2). Therefore, the solidified microstructure of alloy 3C2N consisted of fine, “Chinese-script” Nb(C,N) carbonitride, and the vermicular δ-ferrite (Figure 9e1,e2). (Cr,Fe)_23_C_6_ carbide was not observed in the mushy zone, whereas it precipitated in the following steady-state zone.

#### 3.3.3. Chemical Composition of the Liquid Phase

**Figure 10:**
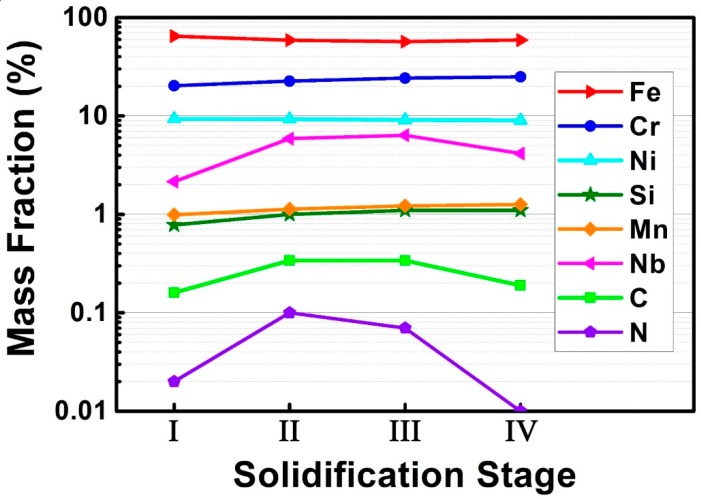
Chemical composition of the liquid phase in different solidification stages marked by red circles in Figure 6b under the withdraw rate of 50 µm/s, quantitatively measured by field emission electron probe microanalyzer (FE-EPMA).

Chemical composition of liquid phase was profoundly associated with the formation mechanism of different phases (γ-austenite, Nb(C,N) and δ-ferrite) in as-cast alloy 3C2N during the LMC-DS process. Figure 10 shows the measured chemical composition of the liquid phase in each solidification stage marked by red circles in Figure 6b at the withdraw rate of 50 µm/s. It is interesting that the contents of Nb, C, and N increased in stage II and III, and then decreased in stage IV, whilst the Cr content increased continuously and significantly until the solidification ended in stage IV. A slight increase in the content of Si and Mn was also noted, rather than Ni and Fe, which the content decreased.

## 4. Discussion

**Figure 11:**
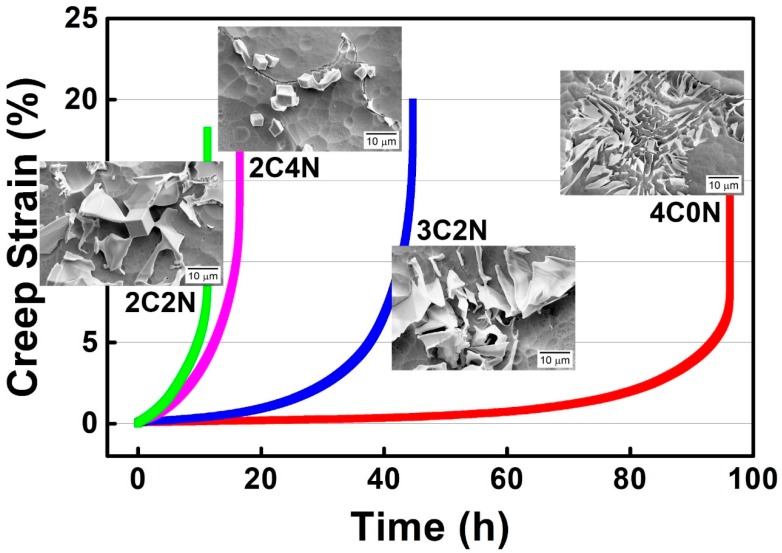
Schematic image showing the relationship between creep property and the microstructure of novel cast austenitic heat-resistant steels at 1000 °C and 50 MPa.

A series of Nb-bearing cast austenitic heat-resistant steels were designed for ultra-high temperature applications in our previous study [7]. Their creep properties have significant microstructural dependence at 1000 °C and 50 MPa, particularly on the morphology of Nb(C,N) carbonitride, as shown in Figure 11. Note that the creep properties of alloys with “Chinese-script” Nb(C,N) carbonitride are much better than that with flake-blocky or facet-blocky Nb(C,N) carbonitride. This is primarily attributed to the “Chinese-script” Nb(C,N) carbonitride that prevents the sliding of grain boundaries at 1000 °C, thus improving the interdendritic strength of these alloys. More interesting, the “Chinese-script” Nb(C,N) carbonitride with finer interlamellar spacing can improve the creep resistance of this type of alloys much more effectively than the coarse carbonitride. It is thus important to elucidate the formation mechanism of Nb(C,N) carbonitride and to grow this fine “Chinese-script” Nb(C,N) carbonitride.

Indeed, the solidification behavior of this type of heat-resistant alloys is profoundly associated with both the chemical composition and the solidification processing. Our previous study reports that these alloys solidify in three modes, depending on the C and N additions: AEF mode (primary austenite + eutectic Nb(C,N) + secondary ferrite), FAEF mode (primary ferrite + secondary austenite + eutectic Nb(C,N) + secondary ferrite) and CAE mode (primary Nb(C,N) carbonitride + primary austenite + eutectic Nb(C,N)) [23]. The microstructural characterization of the mushy zones in as-cast alloy 3C2N demonstrates that the different phases solidified in the order of primary γ-austenite, eutectic Nb(C,N) carbonitride, and then secondary δ-ferrite (Figure 8 and Figure 9), regardless of the withdraw rate. Therefore, the solidification path of alloy 3C2N falls in the AEF mode, and can be described as:

L → L + γ_(1)_ → L + γ_(1)_ + (Nb(C,N) + γ_(2)_)_eutectic_ → L + γ_(1)_ + (Nb(C,N) + γ_(2)_)_eutectic_ + δ → γ_(1)_ + (Nb(C,N) + γ_(2)_)_eutectic_ + δ (3)

This solidification path agrees well with the solidification sequence that is calculated by JMatPro software (Equation (1)), suggesting that it is credible to predict the solidification behavior of these alloys through thermodynamic methods.

Although the solidification path was not changed by increasing the withdraw rate from 20 to 50 µm/s, the eutectic reaction (Equation (2)) range and the PDAS and SDAS, as well as the interlamellar spacing of the “Chinese-script” Nb(C,N) carbonitride were influenced evidently (Figure 6, Figure 8c2 and Figure 9c2). It is generally recognized that the temperature gradient (G_L_) at the L/S interface and the overall withdraw rate (i.e., growth rate, V) play a paramount role in determining the morphology and size of different phases during the LMC-DS process [27]. Since the temperature gradient was kept constant, the withdraw rate is expected to dominate such microstructural alternation.

The eutectic reaction range is expanded, primarily due to the higher withdraw rate of 50 µm/s which induces a larger undercooling. Therefore, austenitic dendrite grows faster at temperatures below the liquidus temperature and a high fraction of solid phase (about 65%) is reached in a shorter time (Figure 7). This contributes to a more rapid accumulation of solutes (Nb, C, and N) rejected from γ-austenite into liquid phase (Figure 10), and enables the elemental concentration to achieve the threshold of eutectic reaction at higher positions of the mushy zone. As a result, the “Chinese-script” Nb(C,N) carbonitride is allowed to form at the distance of 2.6 mm away from the L/S interface through the eutectic approach and the eutectic reaction range is widened accordingly (Figure 6b). It should also be noted that the two peaks on the faction curve withdrawn at 20 µm/s correspond to the formation of floating crystals in stage I, which will remelt at temperatures far beyond the eutectic temperature (Figure 6a and Figure 7). Therefore, these high fractions cannot affect the accumulation of solutes, and thus, the eutectic reaction range.

The PDAS and SDAS are inversely proportional to the G_L_×V product (cooling rate, R) [31]. Actually, plenty of efforts have been paid to investigate the relationship between the PDAS/SDAS and the withdraw rate/cooling rate [32,33,34]. Because the temperature gradient approximates the constant (0.01 °C/µm), the relationship between the PDAS (λ_1_) and the withdraw rate can be described as:

λ_1_ = A × G_L_^−m^ × V^−n^ = B × V^−n^ (4)

where A, B, m, and n are coefficients [31]. Using the PDASs (298.2 and 152.4 µm) at the withdraw rate of 20 and 50 µm/s, B and n are calculated to be 2656 and 0.73, respectively. Therefore, Equation (4) can be modified as:

λ_1_ = 2656 × V^−0.73^ (5)

The values of B and n are comparable to that of a Mg–4Al–4Ca (AX44) alloy during directional solidification reported by Zheng et al. [33].

The relationship between the SDAS (λ_2_) and the withdraw rate/cooling rate is expressed as:

λ_2_ = a × (G_L_ × V)^−x^ = a × R^−x^ = b × V^−x^ (6)

where a, b, and x are coefficients [31]. Using the SDASs (88.8 and 42.3 µm) at the withdraw rate of 20 and 50 µm/s, a, b, and x can be calculated to be 24, 1005, and 0.83, respectively. Accordingly, Equation (6) can be modified as:

λ_2_ = 24 × R^−0.81^ = 1005 × V^−0.81^ (7)

The value of a is comparable to that of conventional steels, whereas the value of x appears to be a bit higher [34]. This might be attributed to there being plenty of alloying elements in alloy 3C2N, particularly the large content of Nb, C, and N, prohibiting the growth of austenitic dendrite. 

The alternation in the interlamellar spacing of the “Chinese-script” Nb(C,N) carbonitride can be attributed to the variations of the G_L_/V ratio. Previous studies have reported that the morphology of solidification growth front changes from planar to cellular, and then to dendritic features with decreasing the G_L_/V ratio [27,31]. Decreasing the withdraw rate from 50 to 20 µm/s increases the G_L_/V ratio, and thus inhibits the growth front to form dendrites. This will retard the formation of “Chinese-script” Nb(C,N) carbonitride, since it prefers to solidify on dendritic interfaces (Figure 8c2 and Figure 9c2). In addition, the relatively low withdraw rate increases the SDAS, and reduces the diffusion rate of solutes between the solid and liquid phases. As a result, the eutectic reaction (equation 2) becomes unstable, through which the Nb(C,N) carbonitride retains the “Chinese-script” morphology, but significantly increases the interlamellar spacing. If the decline of the withdraw rate continues, the Nb(C,N) carbonitride is expected to form faceted and blocky morphologies, without any “Chinese-script” characteristics, similar to that in Ni-based alloys [25]. In contrast, a high withdraw rate facilitates the formation of “Chinese-script” Nb(C,N) carbonitride with small interlamellar spacing, as that in the quenched microstructure (Figure 8a2 and Figure 9a2). It is thus suggested that the withdraw rate is increased for the purpose of fine “Chinese-script” Nb(C,N) carbonitride during solidification.

However, increasing the withdraw rate or the cooling rate should take the macro-grain structure into consideration as well, because fine grains are detrimental to high temperature mechanical properties [7,12,13]. Grains generally decrease with the increase of the withdraw rate or cooling rate (Figure 5), due to the high nucleation rate and insufficient time for the diffusion of solutes [31]. It is thus required to set an upper limit for the withdraw rate or cooling rate of this type of heat-resistant alloys in future study.

Besides the Nb(C,N) carbonitride and the macro-grain structure, the (Cr,Fe)_23_C_6_ carbide affects the ultra-high temperature creep property of these alloys as well. Our previous study recognized that the (Cr,Fe)_23_C_6_ carbide coarsens at 1000 °C, and thus accelerates the nucleation and propagation of creep cracks [12]. Therefore, the quantity of (Cr,Fe)_23_C_6_ carbide should be strictly limited to reduce the adverse effects. The LMC-DS processing confirms that the precipitation of (Cr,Fe)_23_C_6_ carbide is retarded to the steady-state zone of alloy 3C2N [10], indicating that the contents of C and Nb are proper for this alloy design. Therefore, it is of great potential to improve the high temperature mechanical property of alloy 3C2N through increasing the withdraw rate or cooling rate during casting.

## 5. Conclusions

Liquid metal cooling directional solidification of a novel cast austenitic heat-resistant steel was carried out under the withdraw rates of 20 and 50 µm/s. The formation mechanism of Nb(C,N) carbonitride is elucidated, which is helpful for guidance producing fine “Chinese-script” Nb(C,N) carbonitride, and thus improving mechanical properties at ultra-high temperatures. Conclusions are drawn as follows.

1. The solidification path of alloy 3C2N can be described as L → L + γ_(1)_ → L + γ_(1)_ + (Nb(C,N) + γ_(2)_)_eutectic_ → L + γ_(1)_ + (Nb(C,N) + γ_(2)_)_eutectic_ + δ → γ_(1)_ + (Nb(C,N) + γ_(2)_)_eutectic_ + δ, which agrees with the solidification sequence as calculated by thermodynamic methods.
2. The PDAS (λ_1_) and SDAS (λ_2_) are inversely proportional to the withdraw rate (V) and cooling rate (R), and the relationship can be described as λ_1_ = 2656 × V^−0.73^ and λ_2_ = 24 × R^−0.81^ = 1005 × V^−0.81^, respectively.
3. The eutectic reaction range increases with an increase in the withdraw rate, which is primarily attributed to the larger undercooling that induces a rapid increase of the solid phase fraction and an accumulation of solutes in liquid phase.
4. Increasing the withdraw rate facilitates the formation of “Chinese-script” Nb(C,N) carbonitride with small interlamellar spacing, whereas the rate should also be limited for the consideration of preventing fine grains.

## Figures and Tables

**Table 1:** Measured chemical composition of as-cast alloy 3C2N (mass %).

Alloy	Fe	Cr	Ni	Si	Mn	Nb	S	P	C	N
3C2N	Bal.	19.68	10.12	0.80	0.93	2.09	0.008	0.013	0.29	0.15
